# Imaging Characteristics of Embedded Tooth-Associated Cemento-Osseous Dysplasia by Retrospective Study

**DOI:** 10.3390/tomography10020018

**Published:** 2024-02-08

**Authors:** Shun Nishimura, Masafumi Oda, Manabu Habu, Osamu Takahashi, Hiroki Tsurushima, Taishi Otani, Daigo Yoshiga, Nao Wakasugi-Sato, Shinobu Matsumoto-Takeda, Susumu Nishina, Shinji Yoshii, Masaaki Sasaguri, Izumi Yoshioka, Yasuhiro Morimoto

**Affiliations:** 1 Division of Oral and Maxillofacial Radiology, Kyushu Dental University, Kitakyushu 803-8580, Japan; r22nishimura@fa.kyu-dent.ac.jp (S.N.); r07oda@fa.kyu-dent.ac.jp (M.O.); r16wakasugi@fa.kyu-dent.ac.jp (N.W.-S.); r17matsumoto@fa.kyu-dent.ac.jp (S.M.-T.); r22nishina@fa.kyu-dent.ac.jp (S.N.); 2 Division of Maxillofacial Surgery, Kyushu Dental University, Kitakyushu 803-8580, Japan; h-manabu@kyu-dent.ac.jp (M.H.); r07takahashi@fa.kyu-dent.ac.jp (O.T.); r13sasaguri@fa.kyu-dent.ac.jp (M.S.); 3 Division of Oral Medicine, Kyushu Dental University, Kitakyushu 803-8580, Japan; r17tsurushima@fa.kyu-dent.ac.jp (H.T.); r17otani@fa.kyu-dent.ac.jp (T.O.); r11yoshiga@fa.kyu-dent.ac.jp (D.Y.); r13yoshioka@fa.kyu-dent.ac.jp (I.Y.); 4 Division of Promoting Learning Design Education, Department of Physical Function, Kyushu Dental University, Kitakyushu 803-8580, Japan; r08yoshii@fa.kyu-dent.ac.jp

**Keywords:** embedded teeth, panoramic tomograph, CT, cemento-osseous dysplasia

## Abstract

Background: Since there are many differential diagnoses for cemento-osseous dysplasia (COD), it is very difficult for dentists to avoid misdiagnosis. In particular, if COD is related to an embedded tooth, differential diagnosis is difficult. However, there have been no reports on the characteristics of the imaging findings of COD associated with embedded teeth. The aim of the present study was to investigate the occurrence and imaging characteristics of cemento-osseous dysplasia (COD) associated with embedded teeth, in order to appropriately diagnose COD with embedded teeth. Methods: The radiographs with or without histological findings of 225 patients with COD were retrospectively analyzed. A retrospective search through the picture archiving and communication system (PACS) of the Division of Oral and Maxillofacial Radiology of Kyushu Dental University Hospital was performed to identify patients with COD between 2011 and 2022. Results: Fifteen COD-associated embedded mandibular third molars were identified in 13 patients. All 13 patients were asymptomatic. On imaging, COD associated with embedded mandibular third molars appeared as masses that included calcifications around the apex of the tooth. On panoramic tomography, COD showed inconspicuous internal calcification similar to that of odontogenic cysts or simple bone cysts, especially in patients with COD only around the mandibular third molar region. Those with prominent calcification resembled cemento-ossifying fibroma, calcifying epithelial odontogenic tumor, calcifying odontogenic cyst, adenomatoid odontogenic tumor, and so on, as categories of masses that include calcifications on panoramic tomography and computed tomography. Conclusions: The current investigation is the first to report and analyze the imaging characteristics of COD associated with embedded teeth. It is important to consider the differences between COD and other cystic lesions on panoramic tomography, and the differences between COD and masses that include calcifications on CT.

## 1. Introduction

The maxilla and mandible are unique tissues in which teeth erupt, and the diseases that develop in the maxilla and mandible are different from those of other bones. Diseases derived from dental and periodontal origin are unique to the jawbone, and differential diagnosis is important for physicians and dentists. Cemento-osseous dysplasia (COD) is a fibro-osseous lesion (FOL) that represents a reactive process in which normal bone is replaced by cellular fibrous connective tissue and a poorly cellularized cementum-like material [1,2,3,4]. COD is relatively common, but its cause remains unknown [4]. Its origin may be derived from periodontal ligament tissues.

COD is subdivided into periodontal ligament type, focal type, and florid type in the current World Health Organization (WHO) classification [5]. Clinically, COD is usually asymptomatic and is located in the periapical region of teeth with pulp vitality, being commonly identified in routine imaging examinations [6]. COD appears predominantly in Asian middle-aged women [7]. According to representative textbooks, the radiological features of COD include three stages of development: the first is lytic radiolucent, the second is intermediate radiolucency, and the final stage is radiopaque [4,8]. It is difficult to diagnose with imaging because of the variety of imaging findings depending on the stage of the disease. The maxilla and/or mandible are prone to infection in patients with COD, and osteomyelitis tends to occur [3,4]. COD alone is clinically asymptomatic, but dental infection can cause sudden and extensive osteomyelitis. Since there are many differential diagnoses for COD, it is very difficult for dentists to avoid misdiagnosis. In particular, if COD is related to an embedded tooth, differential diagnosis is difficult because it is difficult to differentiate it from tumors or cysts, which may also include calcifications in the maxilla and mandibles. Therefore, it would be extremely important to have an accurate picture of COD, especially of COD that develops on embedded teeth.

To the best of our knowledge, however, there have been no reports of the characteristics of the imaging findings of COD associated with embedded teeth. Appropriate imaging findings for CODs that develop on embedded teeth have not been identified. For the present research purposes, this retrospective study investigates the characteristic imaging findings of COD associated with embedded teeth on panoramic radiographs, multi-detector row computed tomography (MDCT), and cone-beam (CB) CT in order to appropriately diagnose COD with embedded teeth.

## 2. Materials and Methods

In this study, 225 patients with COD (38 males, 187 females; age range 11–92 years; mean age 54.0 ± 17.8 years) were retrospectively analyzed among 16,658 cases using the picture archiving and communication system (PACS) in the Division of Oral and Maxillofacial Radiology of Kyushu Dental University Hospital between 2011 and 2022. The inclusion criteria for COD were the presence of periapical, focal, or florid dysplasia located in the jaws, based on imaging findings according to Cavalcanti et al. [6]. Nine of the COD cases in the present study were evaluated microscopically during pathological examinations after surgical procedures.

Subjects with suspected pathological lesions other than COD, a previous history of craniofacial malformations or syndromes, or a previous history of trauma or surgery were excluded from the present study. Approval of the present study was obtained from the institutional review board of Kyushu Dental University (No. 22-13). The need to obtain informed consent for participation in this study was waived by the review board based on the retrospective design of the study. The informed consent for the publication of the images was obtained from the respective patients. All methods were carried out following the Helsinki Declaration guidelines and regulations. Because COD is often associated with extensive inflammatory changes, many cases were evaluated not only by CBCT but also by MDCT.

Panoramic radiographs were acquired using a panoramic AUTO-1000 EX system (Asahi Roentgen Ind. Co., Ltd., Kyoto, Japan). Images were taken in the incisive occlusion position with the head held by head supports, with the FH plane parallel to the ground. MDCT was performed with an Activion 16 (Toshiba Co. Ltd., Tokyo, Japan). In MDCT imaging, the following parameters for the CT scan were used: 120 kV, 200 mA, 1.0 s per tube rotation, slice thickness of 0.50 mm, helical pitch of 0.688, and table speed of 5.50 mm/rotation. MDCT images were taken with reconstructed images 0.5 mm-thick contiguous sections at the level of the maxilla. The images were obtained with soft-tissue-target windows and bone-target windows by standard algorithms. CBCT was performed with a 3DX (Morita Co. Ltd., Kyoto, Japan) as the CBCT device. In CBCT imaging, the following parameters for the CBCT scan were used: voxel size of 0.16 mm, exposure settings 90 kV and 5 mAs, field of view 40 mm × 40 mm. The CBCT images were converted to volume data with a 0.64 mm slice thickness around the maxilla and mandible, and they were then visualized as axial, panoramic, and sagittal images through multi-planar reconstruction methods.

The imaging characteristics of COD associated with embedded teeth in 225 patients with COD were retrospectively evaluated in the respective teeth on the panoramic radiographs and CT scans (MDCT or CBCT). In detail, the patients were subdivided into periapical, focal, or florid types, based on the WHO classification of COD. In addition, the patients were subdivided into the three textbook stages, first, second, or final stage [4,8]. With respect to the presence of imaging changes in embedded teeth, shape, size, margin, sclerotic rim, internal properties, contiguity between root and internal properties, expansion of cortical bone, and resorption of the mandibular canal were also analyzed. A total of 225 pairs of panoramic tomographs and CT images were assessed by a single, experienced oral and maxillofacial radiologist who had over 10 years of experience and was certified as a specialist by the Japanese Society for Oral and Maxillofacial Radiology (S. N.).

## 3. Results

### 3.1. Distribution of Type Based on the WHO Classification and Stages Based on Imaging Features in Patients with COD

In the present study, 225 of 16,658 cases using a PACS showed COD, for a prevalence of about 1.35%. The distribution of the COD types based on the WHO classification in 225 patients is shown in Table 1. A total of 225 patients with COD were analyzed, of which 13 patients had unerupted teeth with COD. None of the patients classified as having the periapical type had an involved mandibular third molar; 8.8% of patients classified as having the focal type had COD, including a mandibular third molar; and 1.5% of patients classified as having the florid type had COD including a mandibular third molar. In total, 5.8% of all COD patients had an involved mandibular third molar.

The characteristics of the mandibular third molars associated with COD are shown in Table 2. COD around the embedded tooth was seen in 15 lesions of 13 patients. All embedded teeth were mandibular third molars. Of the 15 lesions with COD around the embedded teeth, 13 were focal and 2 were florid. Of the 15 lesions with COD around embedded teeth, 3 were in the first stage, 11 in the second stage, and 1 in the final stage. Seven of 13 patients had COD only around the mandibular third molars.

### 3.2. Imaging Characteristics of COD Associated with Embedded Teeth

Imaging changes around embedded teeth roots were seen in 15 lesions of 13 (Table 2). All embedded teeth with imaging changes related to COD were mandibular third molars. The general visualizations of CODs that developed in the unerupted teeth showed smooth margins and a marginal sclerotic rim. The internal calcification showed dots and uniform calcification, often separated from the root; there was thinning of cortical bone in close proximity to the COD, but little bulging; there was little loss of root associated with the presence of the COD; and there was no loss of root associated with the presence of the COD (Table 2).

**Figure 1:**
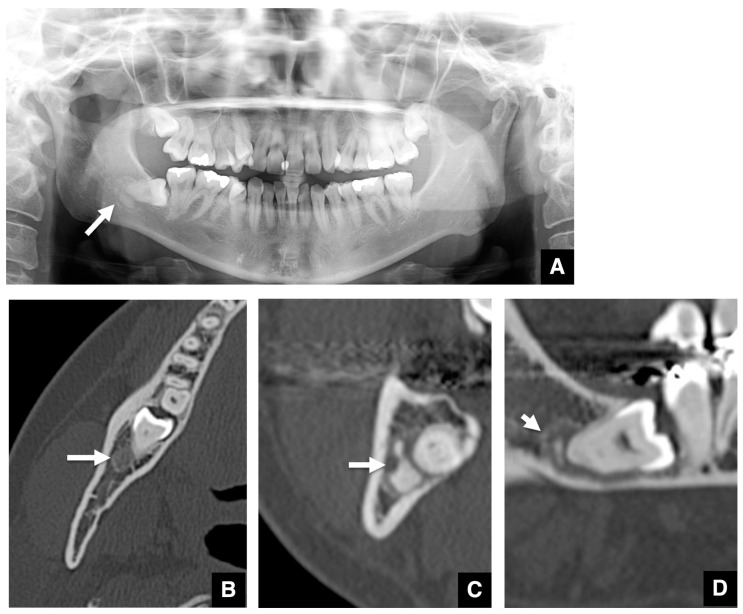
Representative panoramic radiograph and CT images of typical COD related to an embedded mandibular third molar. (**A**) The COD related to an embedded mandibular third molar is visualized as a round area (arrow) with a radiolucent area in the periapical region on the panoramic radiograph. A radiopaque structure within the radiolucent area is visualized. (**B**) The COD related to an embedded mandibular third molar is visualized as a round area (arrow) with low density in the periapical region of the tooth on axial MDCT. The mass with low density includes high-density structures. (**C**) The mass (arrow) and internal high-density structures are shown on coronal MDCT. (**D**) The mass (arrow) and internal high-density structures are shown on sagittal MDCT.

**Figure 2:**
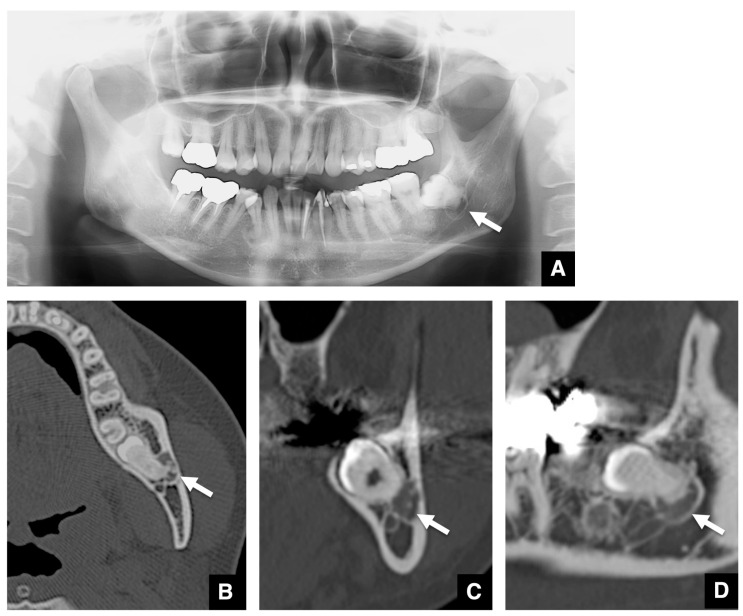
Representative images of COD related to an embedded mandibular third molar with no radiopaque structure identified on the panoramic radiograph. (**A**) The COD related to an embedded mandibular third molar is visualized as a round area (arrow) with a radiolucent area in the periapical region on the panoramic radiograph. The sclerotic rim is clear, and no radiopaque structure is seen. (**B**) The COD related to an embedded mandibular third molar is visualized as a round area (arrow) with low density in the periapical region of the affected tooth on axial MDCT. The low-density mass contains high-density, dot-like structures. The high-density structures are partially contiguous with the roots of the mandibular third molar. There is thinning of the cortical bone adjacent to the lesion. (**C**) The mass (arrow) and internal high-density structures are shown on coronal MDCT. (**D**) The mass (arrow) and internal high-density structures are shown on sagittal MDCT.

**Figure 3:**
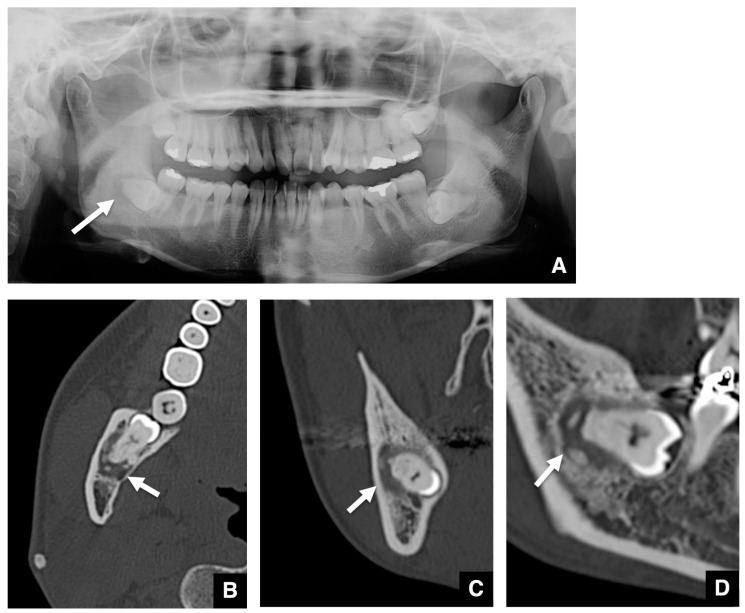
Representative images of COD related to an embedded mandibular third molar with no sclerotic rim seen on the panoramic radiograph. (**A**) The COD related to an embedded mandibular third molar is visualized as a round area (arrow) with a radiolucent area in the periapical region on the panoramic radiograph. The marginal border of the lesion is unclear, and no sclerotic rim is seen. (**B**) The COD related to an embedded mandibular third molar is visualized as a round area (arrow) with low density in the periapical region of the affected tooth on axial MDCT. The high-density structures are partially contiguous with the roots of the mandibular third molar. There is thinning of the cortical bone adjacent to the lesion, and the cortical borders of the mandibular canal tend to disappear. (**C**) The mass (arrow) and internal-high density structures are shown on coronal MDCT. (**D**) The mass (arrow) and internal high-density structures are shown on sagittal MDCT.

The representative features of embedded teeth with imaging changes related to COD are shown in Figure 1, Figure 2 and Figure 3. The COD associated with an embedded mandibular third molar in focal type was visualized as a round area with a radiolucent area in the periapical region on panoramic radiographs (Figure 1A). The margin of the radiolucent area was well-defined and had a sclerotic rim. The radiopaque structure within the radiolucent area was clearly visualized. On axial images of MDCT, the COD associated with an embedded mandibular third molar was visualized as a round area with low density in the periapical (Figure 1B). The low-density mass contained high-density structures. The high-density structures were close, but not contiguous with the roots of the mandibular third molars. The diameter of the mass was about 1 cm. The cortical borders of the mandibular canal tended to disappear. The mass on coronal and sagittal CT images appeared similar to the axial CT image (Figure 1C,D). Other representative images of COD associated with embedded mandibular third molars are shown in Figure 2. COD was visualized as a round radiolucent area around the periapical region on a panoramic radiograph (Figure 2A). The sclerotic rim was clear, and no radiopaque structure was detected. On axial images of MDCT, the COD associated with an embedded mandibular third molar was visualized as a round area with low density in the periapical region (Figure 2B–D). The low-density mass contained high-density, dot-like structures. The high-density structures were partially contiguous with the roots of the mandibular third molars. There was thinning of the cortical bone adjacent to the lesion.

Other imaging features of COD associated with embedded mandibular third molars are shown in Figure 3. COD was visualized as a round radiolucent area around the periapical region on the panoramic radiograph (Figure 3A). The marginal border of the lesion was unclear, and no sclerotic rim was detected. On axial images of MDCT, the COD associated with an embedded mandibular third molar was visualized as a round area with low density in the periapical region (Figure 3B–D). The low-density mass contained high density, dot-like structures. The high-density structures were partially contiguous with the roots of the mandibular third molars. There was thinning of the cortical bone adjacent to the lesion, and the cortical borders of the mandibular canal tended to disappear.

The imaging features on panoramic radiographs and CT images of all CODs associated with embedded mandibular third molars involved in this study are shown in Table 2. The internal properties were visualized in 12 lesions on CT. The internal properties of 3 of the 12 lesions were not visualized on panoramic tomography, even though they were present on CT. In such cases, the features of the masses resembled those of odontogenic cysts or simple bone cysts on panoramic radiographs. In addition, the ill-defined lesions without sclerotic rims resembled bone defects due to rarefying osteitis on panoramic tomography. No resorption of the roots of associated mandibular third molars was seen. The features, such as a low-density mass with high-density structures, clear margin, and expansion, were similar to those of representative benign tumors with internal calcifications.

## 4. Discussion

The present investigation is the first to present and analyze the imaging characteristics of COD associated with embedded teeth. COD appeared as a round area with a radiolucent area in the periapical region, with or without radiopaque internal structures within the area on panoramic radiographs. It is important to differentiate COD from other lesions when cystic lesions are found around the roots of embedded mandibular third molars on panoramic tomographs. On CT, embedded mandibular third molar-related COD was visualized as a round area with low density in the periapical region of the tooth. The features, such as a low-density mass with high-density structures, clear margin, and expansion, were similar to those of representative benign tumors with internal calcifications. In addition, seven of thirteen patients had COD only around the mandibular third molars. It is important for dentists to consider the differences between COD and other masses including calcifications.

The prevalence of COD was from 1.0% to 1.4% in previous reports [9,10,11]. In the present study, 225 of 16,658 cases using a PACS showed COD, for a prevalence of about 1.35%, which was in agreement with previous data [6]. In addition, the patients with COD were predominantly females (83.1%), which was also in agreement with the previous demographic information [6]. These results suggest that the sampling in the present study was quite appropriate.

COD is a relatively common FOL [1,2,3,4]. The cause of COD remains unknown, but it may be derived from periodontal ligament tissues. Pathologically, as the disease progresses, normal bone is gradually replaced by cellular fibrous connective tissue and a poorly cellularized cementum-like material [1,2,3,4]. Therefore, COD should be visualized as a lytic radiolucent area in the first stage, intermediate between radiolucent and radiopaque in the second stage, and finally as a radiopaque area in the third stage during development [4,8]. Despite it being a relatively common disease, to the best of our knowledge, the imaging characteristics of COD around embedded tooth roots have not been described in previous reports and textbooks [1,2,3,4,8]. In the present study, seven patients had COD only around embedded mandibular third molars. The imaging characteristics of COD around the roots of embedded teeth have gone unnoticed by the majority of dentists, including oral and maxillofacial radiologists and surgeons, and they have likely overlooked them. The possible explanations might be inadvertent.

The present investigation is the first to show that the imaging characteristics of COD related to embedded teeth are very similar to those of representative benign tumors, with or without internal calcifications. Particularly, in cases without internal calcifications, it cannot be denied that the absence of calcification, evident in the present study, may lead dentists to assume odontogenic cysts such as a radicular cyst or simple bone cysts, which are frequent occurrences, or an inclusion cyst with an embedded tooth, if they are not appropriately aware of such a case. Therefore, the present study, which has never been reported before, is extremely significant. However, as indicated in the limitations, the study was conducted at a single institution, and the number of cases is limited. The present data should be appropriately applied to embedded teeth with COD of focal type. Therefore, dentists need to be aware of the present results if differential diagnosis of a benign tumor arising at the root apex is required. The present findings indicate that dentists must pay more attention to the imaging characteristics of COD related to embedded teeth, and we expect that many more reports on the changes will be published in the future.

At the same time, the imaging characteristics of COD related to embedded teeth are very similar to those of representative benign tumors with internal calcifications. In concrete terms, the imaging findings of COD with internal calcification in the embedded mandibular third molars were similar to those of cemento-ossifying fibroma (COF), calcifying epithelial odontogenic tumor (CEOT), calcifying odontogenic cyst (COC), adenomatoid odontogenic tumor (AOT), and so on, as categories of masses including calcifications. These diseases are representative examples of masses with calcification in the maxilla and mandible [12,13,14,15]. COF and COD are included among benign fibro-osseous lesions. The radiological appearance of COF was similar to that of COD. Internal calcifications within the mass would increase dependent on the maturity of the lesion, as mentioned above. AOTs refer to epithelial odontogenic tumors and are characterized by a mass surrounding the crown of an unerupted tooth that contains sporadic calcified material inside, as indicated by imaging characteristics [12,13]. The characteristics of AOT are similar to those of COD. CEOT is another extremely rare epithelial odontogenic tumor, and the imaging characteristics of CEOT show similarities to those of COD, presenting as masses with internal calcification [14]. COC is an odontogenic cyst, and the imaging characteristics were visualized as masses with internal calcification [15]. When a solitary COD develops on an embedded tooth, it is difficult to make a clear differential diagnosis by imaging between it and an COF, AOT, CEOT, or COC that develops on the root apex of an embedded tooth. Therefore, when we find a calcified mass in the root apex of an embedded tooth, it is important to consider the possibility of COF, AOT, CEOT, or COC in addition to COD before making a differential diagnosis. A recently published paper on CEOT also showed that COF, AOT, and COD have similar characteristics, including demographics, clinical presentation, and radiographic features [14]. Similar characteristics are also observed for CODs that occur in embedded teeth. In such cases, it is important to differentiate COD based on the fact that it is likely to be multiple, and that the mass covers most of the root apex. Furthermore, COD with embedded teeth is not so different from COD with erupted teeth.

At the same time, dentists should consider the increased risk of infection of the bone. The bone tissue affected by COD is more susceptible to infections due to unknown causes, and invasive procedures such as dental minor surgeries could cause difficulties [16]. In particular, dentists must pay attention to medication-related osteonecrosis of the jaws (MRONJ), which has recently emerged as a serious side effect in bisphosphonate-treated patients exhibiting prominent bone-destructive changes, including osteonecrosis. Of course, it has been suggested that bone tissue affected by MRONJ is more susceptible to infection in patients with COD because COD tends to have poor vascularization [17]. The most important issue to keep in mind should be to prevent dental infections in patients with COD.

What was surprising about the results of this study was that a high percentage of cases with COD in the mandibular third molars showed partial loss of the mandibular canal wall running around the lesion. In other words, there was a high probability of contact between the lesion and the mandibular canal. In this case, the possibility of induction of inferior alveolar nerve damage is increased when the mandibular third molar is extracted, and the lesion is removed. Of course, contact between the mandibular third molar and the mandibular canal is often encountered, even in the absence of a lesion, but when the disease is involved, caution should be exercised during the extraction investigation. Similarly, in the presence of a lesion, thinning or loss of the buccolingual cortical bone of the mandible adjacent to the lesion was found in a high percentage of cases. This condition also suggests a little increase of the possibility of perforation or fracture of the buccolingual cortical bone when the mandibular third molar is extracted, and the lesion is removed. This means an increased likelihood of inducing inflammatory changes to the soft tissues after the procedure. Therefore, extraction of mandibular third molars with COD should be performed with the utmost caution and after careful analysis of the images.

The important limitation of the present study is that the pathological diagnosis for the patients with COD could not be determined. The patients with COD in the present report could be only selected by having representative imaging findings. COD patients without typical imaging findings might have been excluded. At the same time, hidden imaging findings, other than the characteristic ones, might be present in patients with COD. However, the definitive diagnosis of COD is not always made by pathology and is rarely made clinically. This limitation is obviously difficult to resolve because of ethical issues. Moreover, other limitations include that the sample size was not very large, and all patients were Japanese.

## 5. Conclusions

The occurrence and imaging characteristics of COD around embedded teeth was investigated, and the radiographic and/or histological findings of 225 patients with COD were retrospectively analyzed from 16,658 cases through PACS of the Division of Oral and Maxillofacial Radiology of Kyushu Dental University Hospital. The prevalence of COD was about 1.35%. Thirteen patients were found to have COD in embedded mandibular third molars, with no symptoms. The imaging characteristics observed in all cases of COD associated with embedded mandibular third molars included cystic masses, with or without calcifications, around the apex of a tooth. General visualizations of CODs that developed in unerupted teeth showed smooth margins and a marginal sclerotic rim. The internal calcification showed dots and uniform calcification, often separated from the root; there was thinning of cortical bone in close proximity to the COD, but little bulging; there was little loss of root associated with the presence of the COD; and there was no loss of root associated with the presence of COD. The imaging findings of COD in embedded mandibular third molars, without internal calcification on panoramic tomographs, were similar to those of odontogenic cysts or simple bone cysts. The imaging findings of COD with internal calcification in the embedded mandibular third molars were similar to those of cemento-ossifying fibroma, calcifying epithelial odontogenic tumor, calcifying odontogenic cyst, adenomatoid odontogenic tumor, and so on, as categories of masses that include calcifications. The present investigation is the first to present and analyze the imaging characteristics of COD around embedded teeth. It is important to consider the differences between COD and masses that include calcifications.

## 6. Clinical Significance

The present investigation is the first to present and analyze the imaging characteristics of COD associated with embedded teeth. It is important to consider the differences between COD and other lesions.

## Figures and Tables

**Table 1:** Distribution of COD type based on the WHO classification in patients with COD.

WHO Classification	Number of Patients	Number of Lesions Including the Mandibular Third Molar	Rate (%)
Periapical	23	-	-
Focal	137	12	8.8
Florid	65	1	1.5
Total	225	13	5.8

**Table 2:** Characteristic imaging changes associated with embedded mandibular third molars.

								On Panoramic Tomography			On CT Imaging						
Age (y)	Sex	Side	WHO Classification	Stage	Shape	Size (mm)	Impaction of Associated Mandibular Third Molar	Margin	Sclerotic Rim	Internal Properties	Margin	Sclerotic Rim	Internal Properties	Contiguous between Root and Internal Properties	Thinning of Adjacent Cortical Bone	Expansion of Cortical Bone	Resorption of Mandibular Canal
48	F	Left	Focal	Second	Round	7.0 × 4.3 × 7.1	Horizontal	Well-defined	Thin	Undetectable	Smooth	Present	Radiopaque dots	Distinct	Present	Present	Absent
33	F	Right	Focal	Second	Round	8.6 × 7.0 × 7.2	Horizontal	Well-defined	Thick	Crescent	Not smooth	Present	Uniform calcification	Distinct	Present	Absent	Absent
		Left	Focal	Second	Round	7.9 × 7.3 × 7.1	Horizontal	Well-defined	Undetectable	Undetectable	Smooth	Present	Radiopaque dots	Partial contact	Present	Absent	Present
35	F	Right	Focal	First	Round	9.1 × 8.6 × 10.4	Horizontal	Ill-defined	Thin	Undetectable	Smooth	Absent	Undetectable	Distinct	Present	Absent	Present
39	F	Right	Focal	Second	Round	14.8 × 8.5 × 9.9	Horizontal	Well-defined	Undetectable	Crescent	Smooth	Present	Uniform calcification	Distinct	Absent	Absent	Present
47	F	Right	Focal	Second	Irregular	8.4 × 6.0 × 9.7	Vertical	Well-defined	Undetectable	Pearls	Not smooth	Absent	Massive calcification	Partial contact	Present	Absent	Present
42	F	Left	Focal	Second	Irregular	10.2 × 5.6 × 13.0	Horizontal	Well-defined	Undetectable	Multiple dots	Smooth	Present	Radiopaque dots	Distinct	Present	Absent	Present
51	F	Right	Focal	Second	Round	9.8 × 6.2 × 5.8	Horizontal	Ill-defined	Undetectable	Crescent	Smooth	Present	Uniform calcification	Partial contact	Absent	Absent	Present
33	F	Left	Focal	Second	Round	10.3 × 8.7 × 11.5	Vertical	Ill-defined	Undetectable	Multiple dots	Smooth	Present	Massive calcification	Contiguous	Present	Absent	Present
62	M	Right	Focal	First	Round	6.0 × 2.9 × 5.7	Horizontal	Well-defined	Undetectable	Undetectable	Smooth	Present	Undetectable	Distinct	Absent	Absent	Absent
44	M	Left	Focal	First	Round	8.7 × 5.7 × 5.8	Horizontal	Ill-defined	Undetectable	Undetectable	Smooth	Absent	Undetectable	Distinct	Absent	Absent	Absent
45	F	Right	Florid	Second	Round	11.9 × 8.6 × 11.7	Horizontal	Ill-defined	Undetectable	Undetectable	Smooth	Present	Multiple radiopaque dots	Partially contact	Absent	Absent	Present
		Left	Florid	Second	Round	10.2 × 5.6 × 11.9	Horizontal	Ill-defined	Thin	Multiple dots	Not smooth	Present	Multiple radiopaque dots	Partially contact	Present	Absent	Present
38	F	Left	Focal	Final	Round	13.2 × 9.8 × 10.2	Horizontal	Well-defined	Thick	Pearls	Not smooth	Absent	Uniform calcification	Contiguous	Present	Absent	Absent
35	F	Left	Focal	Second	Round	11.7 × 7.1 × 10.0	Horizontal	Well-defined	Thin	Multiple dots	Smooth	Present	Radiopaque dots	Partial contact	Present	Absent	Present

## Data Availability

For confidentiality issues, the data will only be shared in aggregate form as presented in the figures and tables.
